# Chronic Thromboembolic Pulmonary Hypertension as *RNF213*-Associated Vascular Disease

**DOI:** 10.1016/j.jacasi.2024.09.012

**Published:** 2024-11-19

**Authors:** Mizuki Momoi, Yoshinori Katsumata, Takahiro Hiraide, Yoshiki Shinya, Shinichi Goto, Takumi Inami, Kenjiro Kosaki, Masaharu Kataoka

**Affiliations:** a Department of Cardiology, Keio University School of Medicine, Tokyo, Japan; b Institute for Integrated Sports Medicine, Keio University School of Medicine, Tokyo, Japan; c Division of Cardiovascular Medicine, Brigham and Women’s Hospital, Boston, Massachusetts, USA; d Harvard Medical School, Boston, USA; e Division of General Internal Medicine and Family Medicine, Department of General and Acute Medicine, Tokai University School of Medicine, Isehara, Japan; f Department of Cardiovascular Medicine, Kyorin University School of Medicine, Tokyo, Japan; g Center for Medical Genetics, Keio University School of Medicine, Tokyo, Japan; h Second Department of Internal Medicine, University of Occupational and Environmental Health, Kitakyushu, Japan

**Keywords:** balloon pulmonary angioplasty, chronic thromboembolic pulmonary hypertension, peripheral pulmonary artery stenosis, poor subpleural perfusion, *RNF213*

Although some risk factors for chronic thromboembolic pulmonary hypertension (CTEPH) have been reported,^1^ many patients do not present with these risk factors. Moreover, the pathogenesis of CTEPH has not been fully elucidated. Recently, a variant of c.14429G>A (p.R4810K, rs112735431) in the ring finger protein 213 gene (*RNF213*) was reported to be associated with systemic vasculopathy, named “*RNF213*-associated vascular disease,” which includes moyamoya disease, pulmonary arterial hypertension, and peripheral pulmonary artery stenosis (PPS).^2^ However, the prevalence and clinical impact of the *RNF213* p.R4810K variant in patients with CTEPH have not been investigated. Therefore, we analyzed the *RNF213* p.R4810K variant in patients with CTEPH.

This study was performed at 2 university hospitals in Japan. The study was approved by the institutional ethics committees (approval numbers: 20140203 and H26-125), and written informed consent was obtained from all participants. Patients with CTEPH who underwent balloon pulmonary angioplasty (BPA) between 2009 and 2021 were included. CTEPH was diagnosed according to the current guidelines. Patients with strong or moderate risk factors for venous thromboembolism, except for previous venous thromboembolism, as defined in a previous report,^1^ were excluded from the analysis. Whole-exome sequencing was performed for the genetic analysis. Data were collected at diagnosis, including World Health Organization functional class, blood tests, medications, and hemodynamics measured using right heart catheterization. To assess treatment response, follow-up data were collected at the time of follow-up right heart catheterization 1 year after the completion of BPA. The clinical parameters were compared between patients with and without the *RNF213* p.R4810K heterozygous variant at both time points. Multivariate analysis was also performed to assess the impact of the *RNF213* p.R4810K heterozygous variant on the treatment response after adjustment of disease severity at diagnosis.

A total of 179 patients with CTEPH were included in this study. Of these, 39 patients who had known risk factors for venous thromboembolism were excluded. Thus, 140 patients with CTEPH were analyzed and the *RNF213* p.R4810K heterozygous variant was confirmed in 9 (6.4%) patients. The allele frequency was 0.0321, whereas it is 0.0084 in the general Japanese population according to the integrative Japanese genome variation database, the genome cohort study of the Tohoku Medical Megabank Organization. The other 18 pathogenic or likely pathogenic variants in *RNF213*, which are registered in the latest ClinVar database,^3^ were not detected. The patient characteristics are summarized in Table 1. Patients with the *RNF213* p.R4810K variant were significantly younger and had higher mean pulmonary arterial pressure (PAP) at diagnosis than those without the variant. Regarding angiographic findings, no patients presented the findings of PPS such as elongated stenosis and string of beads pattern. The distribution of thrombi assessed by the surgical classification did not differ between patients with and without *RNF213* p.R4810K heterozygous variant. However, patients with this variant exhibit high frequency of poor subpleural perfusion, which has been associated with poor treatment response.^4^ With respect to the treatment response during the median follow-up period of 632 days (Q1-Q3: 502-845 days) after diagnosis, the mean PAP and pulmonary vascular resistance (PVR) at follow-up were significantly higher in patients with the variant than in those without the variant (Table 1). After adjustment with multiple linear regression analysis using age, sex, B-type natriuretic peptide, World Health Organization functional class, PVR, cardiac output, 6-minute walk distance, and SvO_2_ at diagnosis as a covariate, this variant was significantly associated with high PVR at follow-up (β = 1.210; 95% CI: 0.137-2.283; *P =* 0.027). Furthermore, the number of BPA treatment sessions was greater in patients with the variant (average: 5.18 vs 7.33 times, respectively; *P =* 0.011) than in those without the variant. This was also confirmed after adjustment with multiple linear regression analysis (β = 1.795; 95% CI: 0.340-3.251; *P =* 0.016).

**Table 1:** Characteristics and Treatment Response of CTEPH Patients With and Without *RNF213* p.R4810K Variant

	All (N = 140)	Missing Values, n	*RNF213* R4810K− (n = 131)	Missing Values, n	*RNF213* R4810K+ (n = 9)	Missing Values, n	*P* Value
Baseline characteristics							
Female	105 (75.0)	0	99 (75.6)	0	6 (66.7)	0	0.84
Age at diagnosis, y	66 (56-73)	0	67 (56-73)	0	56 (46-66)	0	0.032
History of DVT/PE	64 (45.7)	0	58 (44.3)	0	6 (66.7)	0	0.34
eGFR, mL/min/1.73 m^2^	62 (47-73)	2	62 (47-73)	2	57 (51-63)	0	0.73
BNP, pg/mL	76 (27-287)	1	76 (27-320)	1	151 (35-169)	0	0.87
WHO-FC of I/II/III/IV	3/29/97/5 (2.2/21.6/72.4/3.7)	6	3/28/89/5 (2.4/22.4/71.2/4.0)	6	0/1/8/0 (0.0/11.1/88.9/0.0)	0	0.70
Medication							
Anticoagulation drugs	140 (100)	0	131 (100)	0	9 (100)	0	NA
Soluble guanylyl cyclase	13 (9.3)	0	12 (9.2)	0	1 (11.1)	0	1.0
PDE 5 inhibitors	55 (39.3)	0	51 (38.9)	0	4 (44.4)	0	1.0
Oral prostacyclin analogs	41 (29.3)	0	40 (30.5)	0	1 (11.1)	0	0.39
Endothelin receptor antagonist	50 (35.7)	0	47 (35.9)	0	3 (33.3)	0	1.0
Hemodynamics							
Mean RAP, mm Hg	5 (2-7)	1	5 (2-7)	1	7 (3-9)	0	0.36
Mean PAP, mm Hg	38 (31-45)	0	38 (31-45)	0	47 (41-51)	0	0.035
PAWP, mm Hg	8 (6-10)	2	8 (6-10)	2	9 (7-10)	0	0.37
CO, L/min	3.74 (3.00-4.44)	2	3.73 (2.95-4.40)	2	4.05 (3.64-5.20)	0	0.29
PVR, WUs	7.99 (5.64-12.97)	2	7.87 (5.56-12.98)	2	9.90 (6.77-10.82)	0	0.65
Surgical classification of I/II/III/IV	0/31/61/48 (0/22.1/43.6/34.3)	0	0/29/57/45 (0.0/22.1/43.5/34.4)	0	0/2/4/3 (0.0/22.2/44.4/33.3)	0	1.0
PSP	18 (12.9)	1	12 (9.2)	1	6 (66.7)	0	<0.001
HOT	97 (70.8)	2	90 (70.3)	2	7 (77.8)	0	0.92
6MWD, m	375 (280-430)	11	377 (284-437)	10	321 (250-417)	1	0.51
Treatment strategy							
PEA	10 (7.1)	0	9 (6.9)	0	1 (11.1)	0	1.0
Follow-up data							
BNP, pg/mL	25 (14-47)	1	25 (14-46)	1	35 (9-49)	0	0.80
Hemodynamics							
Mean RAP, mm Hg	3 (1-4)	1	3 (1-4)	1	2 (2-3)	0	0.89
Mean PAP, mm Hg	20 (16-22)	1	19 (16-22)	1	22 (20-35)	0	0.045
PAWP, mm Hg	7 (5-10)	1	7 (5-10)	1	8 (5-10)	0	0.91
CO, L/min	4.39 (3.62-5.22)	1	4.34 (3.61-5.20)	1	4.66 (4.18-5.50)	0	0.21
PVR, WU	2.75 (1.91-3.57)	1	2.62 (1.88-3.35)	1	3.89 (3.09-5.14)	0	0.040
HOT	43 (30.9)	1	39 (30.0)	1	4 (44.4)	0	0.59
6MWD, m	429 (371-505)	11	429 (373-506)	10	429 (263-459)	1	0.39
WHO-FC of I/II/III/IV	56/81/2/0 (40.3/58.3/1.4/0)	1	54/75/1/0 (41.5/57.7/0.8/0)	1	2/6/1/0 (22.2/66.7/11.1/0)	0	0.028

Values are n (%) or median (Q1-Q3). *P* values from the Mann-Whitney *U* test for continuous data and the chi-square or Fisher exact test for categorical data, as appropriate, are displayed.

6MWD = 6-minute walk distance; BNP = B-type natriuretic peptide; CO = cardiac output; DVT = deep vein thrombosis; HOT = home oxygen therapy; PAP = pulmonary arterial pressure; PAWP = pulmonary arterial wedge pressure; PE = pulmonary embolism; PEA = pulmonary endarterectomy; PSP = poor subpleural perfusion; PVR = pulmonary vascular resistance; RAP = right atrial pressure; RBC = red blood cell; *RNF213* = ring finger protein 213; WBC = white blood cell; WHO-FC = World Health Organization functional class.

The *RNF213* p.R4810K heterozygous variant has been reported not only in patients with moyamoya disease, but also in approximately 8% of patients diagnosed with idiopathic pulmonary arterial hypertension.^2^ Moreover, a previous study reported that *RNF213* p.R4810K homozygous variants were confirmed in patients with PPS.^5^ Thus, a novel concept of “*RNF213*-associated vascular disease” was revealed.^2^ Importantly, this study demonstrated that some patients with *RNF213*-associated vascular diseases were also diagnosed with CTEPH. Hence, these findings suggest the potential for patients with *RNF213*-associated vascular disease primarily affecting pulmonary capillary levels to be diagnosed with pulmonary arterial hypertension. Conversely, patients with involvement at more proximal levels may be diagnosed with CTEPH, given the presence of secondary organized thrombi, or with PPS in cases of homozygous variants.

This study also confirmed poor treatment response after BPA in patients with *RNF213* p.R4810K variant diagnosed with CTEPH. This may be caused by recoil or restenosis in response to BPA treatment caused by abnormalities in the vascular intima, as observed in PPS.^6^ The high frequency of poor subpleural perfusion in patients with the *RNF213* p.R4810K heterozygous variant also suggests the possibility that this variant promotes the pathological changes in the pulmonary arterial small vessel walls, which could be an important angiographic finding in CTEPH patients with this variant. However, these findings must be confirmed using intravascular imaging and pathology.

In conclusion, the *RNF213* p.R4810K heterozygous variant was identified in 9 (6.4%) patients with CTEPH without known risk factors for venous thromboembolism, suggesting that some patients diagnosed with CTEPH have *RNF213*-associated vascular disease, which is associated with poor treatment response after BPA. Genetic testing of the *RNF213* variant is crucial and may lead to precision medicine based on genetics, even in patients with CTEPH. Further investigation in a larger cohort is needed to test our findings and the pathogenicity of the *RNF213* p.R4810K variant in patients with CTEPH.

## Funding Support and Author Disclosures

This research was supported by the Japan Agency for Medical Research and Development (AMED) under Grant Number JP23ek0109535. The authors have reported that they have no relationships relevant to the contents of this paper to disclose.
